# Supplementary material for: Drug repurposing for aging research using model organisms
Source: Aging Cell. 2017 Jun 16;16(5):1006–15. doi: 10.1111/acel.12626 (PMC5595691; doi:10.1111/acel.12626)
Supplement: Supplementary file 7 — Data S1 Zip‐Archive of all report cards. [file ACEL-16-1006-s007.zip › RC_SAL.pdf]

SAL

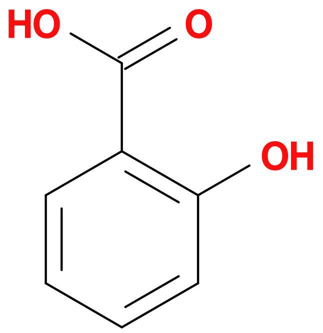

Database identifiers

ChEMBLCompound	CHEMBL424
DrugBank	DB00936
CHEBI	16914
eMolecules	478035

Ranking

	Rank	Score
Drosophila	330/697	0.469
C. elegans	204/591	0.245

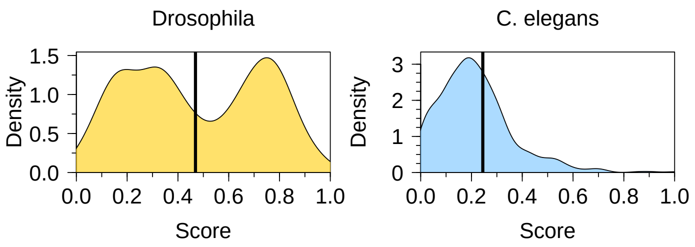

	Ageing implication	Domain conservation	Binding site conservation	Binding affinity	Bioavailability	Lipinski	Promiscuity	Purchasability	Drug approval	**Total**
Drosophila	1.0	0.932	0.908	0.386	(0.9)	0.0	-0.0	0.1	0.075	0.469
C. elegans	1.0	0.915	0.859	0.386	0.231	0.0	-0.0	0.1	0.075	0.245

Names

- **salicylic acid**
- Advanced Pain Relief Callus Removers
- Advanced Pain Relief Com Removers
- Clear Away Wart Remover
- Compound W
- Dr. Scholl's Callus Removers
- Dr. Scholl's Corn Removers
- Dr. Scholl's Wart Remover Kit
- Duofilm Wart Remover
- Duoplant
- Freezone
- Ionil

· Ionil-Plus
· o-carboxyphenol
· o-Hydroxybenzoic acid
· Saligel
· Stri-Dex

Roles

plant metabolite, EC 1.11.1.11 (L-ascorbate peroxidase) inhibitors, antiinfective agent, antifungal agent, keratolytic drug, algal metabolite, plant hormone

Status

Approved drug (according to ChEMBL)	No
Classification (according to DrugBank)	approved
Administration Route	Topical, Intravenous, Oral
Number of Rule of 5 violations	0
Binding affinity to original target in log units (RF-Score prediction)	4.54
Burns *C. elegans* bioavailability prediction	-3.25

Medical Information

Indicatation: Key additive in many skin-care products for the treatment of acne, psoriasis, callouses, corns, keratosis pilaris and warts.
Mechanism of action: Salicylic acid directly and irreversibly inhibits the activity of both types of cyclo-oxygenases (COX-1 and COX-2) to decrease the formation of precursors of prostaglandins and thromboxanes from arachidonic acid. Salicylate may competitively inhibit prostaglandin formation. Salicylate's antirheumatic (nonsteroidal anti-inflammatory) actions are a result of its analgesic and anti-inflammatory mechanisms. Salicylic acid is a key ingredient in many skin-care products for the treatment of acne, psoriasis, calluses, corns, keratosis pilaris, and warts. It works by causing the cells of the epidermis to slough off more readily, preventing pores from clogging up, and allowing room for new cell growth. Because of its effect on skin cells, salicylic acid is used in several shampoos used to treat dandruff. Salicylic acid is also used as an active ingredient in gels which remove verrucas (plantar warts). Salicylic acid inhibits the oxidation of uridine-5-diphosphoglucose (UDPG) competitively with nicotinamide adenosine dinucleotide (NAD) and noncompetitively with UDPG. It also competitively inhibits the transferring of glucuronyl group of uridine-5-phosphoglucuronic acid (UDPGA) to the phenolic acceptor. The wound-healing retardation action of salicylates is probably due mainly to its inhibitory action on mucopolysaccharide synthesis.
Toxicity: Oral rat LD50: 891 mg/kg. Inhalation rat LC50: $>$ 900 mg/m3/1hr. Irritation: skin rabbit: 500 mg/24H mild. Eye rabbit: 100 mg severe. Investigated a mutagen and reproductive effector.
Retail: 7 Oaks Pharmaceutical Corp., Aeropak (Uk), Auriga Pharmaceuticals LLC, Avidas Pharmaceuticals, Breckenridge Pharmaceuticals, Collegium Pharmaceutical Inc. and 36 more suppliers
Salicylic acid 6% gel - 1.14 USD / g
Sastid soap - 4.32 USD / each
Keralyt 3% Gel 28.4 gm Tube - 11.35 USD / tube
Compound W 17% Liquid 9.3ml Bottle - 11.99 USD / bottle
Keralyt 6% Gel 40 gm Tube - 49.99 USD / tube
Keralyt 6% Gel 100 gm Tube - 51.99 USD / tube
and 10 more options

(Information from DrugBank)

Compound Target Characteristics

Xanthine dehydrogenase/oxidase

Best gene implication in ageing for this target family came from gene P10351 annotated in UniProt release 2014_02. Annotation GO 8340 (determination of adult lifespan) was Inferred from Mutant Phenotype

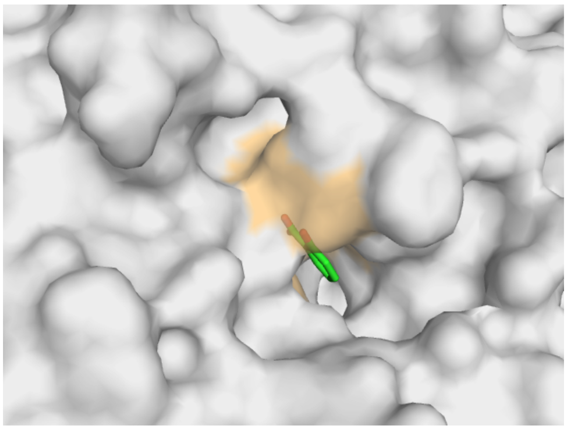

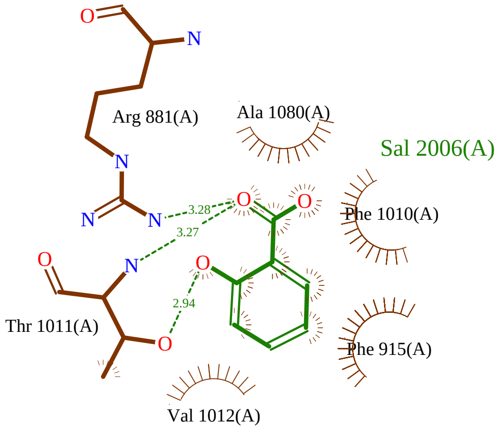

protein	amino acids contacts (binding site)					
PDB:2e1q:chainA:P47989	R	F	F	T	V	A
sp:P47989:XDH_HUMAN	R	F	F	T	V	A
tr:F1LQS6:F1LQS6_RAT	R	F	F	T	L	A
sp:Q00519:XDH_MOUSE	R	F	F	T	L	A
sp:P10351:XDH_DROME	R	F	F	G	V	A
tr:O17892:O17892_CAEEL	R	F	F	G	L	A

	whole protein		domain-based		contact-based	
protein	ident	simil	ident	simil	ident	simil
PDB:2e1q:chainA:P47989	1.0	1.0	1.0	1.0	1.0	1.0
sp:P47989:XDH_HUMAN	1.0	1.0	1.0	1.0	1.0	1.0
tr:F1LQS6:F1LQS6_RAT	0.9	0.97	0.89	0.97	0.83	0.95
sp:Q00519:XDH_MOUSE	0.89	0.97	0.88	0.97	0.83	0.95
sp:P10351:XDH_DROME	0.52	0.83	0.58	0.86	0.83	0.91
tr:O17892:O17892_CAEEL	0.46	0.78	0.54	0.84	0.67	0.86

ry (FBgn0003308) associated phenotypes

chemical conditional, chemical sensitive, heat sensitive, partially, partially lethal, partially lethal - majority die, radiation sensitive, short lived

(Information from FlyBase)

ry (UniProt:P10351) annotation

Function: Key enzyme in purine degradation. Catalyzes the oxidation of hypoxanthine to xanthine. Catalyzes the oxidation of xanthine to uric acid (By similarity).
Cofactor: [2Fe-2S] cluster Evidence=; Note=Binds 2 [2Fe-2S] clusters. ;
Cofactor: FAD
Cofactor: Mo-molybdopterin Evidence=; Note=Binds 1 Mo-molybdopterin (Mo-MPT) cofactor per subunit. ;
Subunit: Homodimer.
Subcellular location: Peroxisome

(Information from UniProt)
